# Clinical and radiographic features of pycnodysostosis: A case report

**DOI:** 10.4317/jced.54105

**Published:** 2017-10-01

**Authors:** Cleomar Rodrigues, Fernando-Antônio Gomes, José-Alcides Arruda, Luciano Silva, Pâmella Álvares, Priscila da Fonte, Ana-Paula Sobral, Marcia Silveira

**Affiliations:** 1 School of Dentistry, Faculdades Integradas da União Educacional do Planalto Central (FACIPLAC), Brasília, DF, Brazil; 2 Brazilian Association of Dentistry, ABO-Brasilia, Brazil; 3 Department of Oral and Maxillofacial Pathology, School of Dentistry, Universidade de Pernambuco, Recife, PE, Brazil; 4 Department of Orthodontics, School of Dentistry, Universidade de Pernambuco, Recife, PE, Brazil; 5 Department of Oral Diagnosis, School of Dentistry, Universidade de Pernambuco, Recife, PE, Brazil

## Abstract

Pycnodysostosis is a rare disorder that was first described in 1962; however, it was only in 1996 that the defective gene was discovered, which led to a better understanding of this disease. This study reports and discuss a case of pycnodysostosis. In addition, a search of articles published in PubMed-Medline was performed. The case was a 13-year-old girl who was referred to a private clinic for dental treatment. Clinical examination showed midface hypoplasia, prominent cheeks, a high nasal bridge, beaked nose, spoon-shaped fingers, frontal bossing, open fontanelles and dental alterations, findings compatible with pycnodysostosis. Patients with this disease also suffer from fractures because of bone hardness with almost no elasticity, a fact that requires special care particularly in the case of children and adolescents. The diagnosis of pycnodysostosis is made based on clinical and radiographic findings. Clinicians should be aware of this disorder to provide adequate dental treatment.

** Key words:**Pycnodysostosis, developmental bone disease, imaging diagnosis.

## Introduction

Dysostosis are defined as malformations that affect single or multiple bones in combination and their existence is due to abnormal blastogenesis in utero which alters the phenotype of the bearers, giving particular characteristics that may be observed clinically to enhance diagnosis (1). Among them, pycnodysostosis (PYCD) can be described as a disorder that exhibits many alterations in many different parts of the body, with special features in the orofacial region. Statistically, there is an estimated prevalence of 1 to 1.7 per million births based on the number of cases described in the literature worldwide (2,3). A striking fact that made PYCD known in the academic world was that the French painter Henri de Toulouse-Lautrec who was affected by it, a fact that made this dysostosis be first named Toulouse-Lautrec syndrome (2). Although first described in 1962, it was only in 1996 that the defective gene encoding cathepsin K (CTSK) was identified, offering the opportunity of a more accurate diagnosis for patients whose clinical and radiographic features were doubtful (4,5). The absence of this enzyme implies that osteoclast can not resorb the organic matrix properly, impairing bone remodeling, and therefore being responsible for fragile and dense bones as a consequence of such insufficient bone resorption (4,5).

The diagnosis of PYCD should be established systematically based on clinical aspects complemented by imaginological examinations, and often require radiographs, tomography and magnetic resonance (6,7). The main characteristics of PYCD are terminal phalanges of hands and toes with drumstick appearance. In the face, a midface hypoplasia, prominent cheeks, high nasal bridge, frontal bossing, beaked nose, projection or retraction of the mandible, and micrognathia associated with malocclusion such as crossbite and open bite (6-9). Dental anomalies, enamel hypoplasia, hypercementosis, narrowing of the pulp chamber and root canals, erosion of alveolar crest without clinical evidence of periodontitis, are generally associated with poor oral hygiene in patients with PYCD (6-10). Platybasia, a spinal disease of a malformed relationship between the occipital bone and cervical spine has been described in some patients, not necessarily associated with other clinical features such as narrow or drooping shoulders, and sometimes difficulty in hearing (2,6,10). Facial asymmetry and micrognathia have also been frequently reported.

Although PYCD has been known and studied for almost 50 years, there are only few case reports describing oral and maxillofacial features. This study describes the case of a 13-year-old Brazilian girl with PYCD, associating clinical, radiographic and tomographic features. In addition, a Medline search comprising the period from 1962 to March 2017 was performed and cases of PYCD around the world were mapped.

## Case Report

A 13-year-old Brazilian girl was referred to a private clinic for dental treatment. During clinical examination, it was noted that the patient had a short stature (128 cm) and weight of 30 kg. During anamnesis, the mother reported no intercurrences during pregnancy or in neonatal history. The girl had a history of recurrent accidents due to lower limb fractures during childhood.

**Figure 1:**
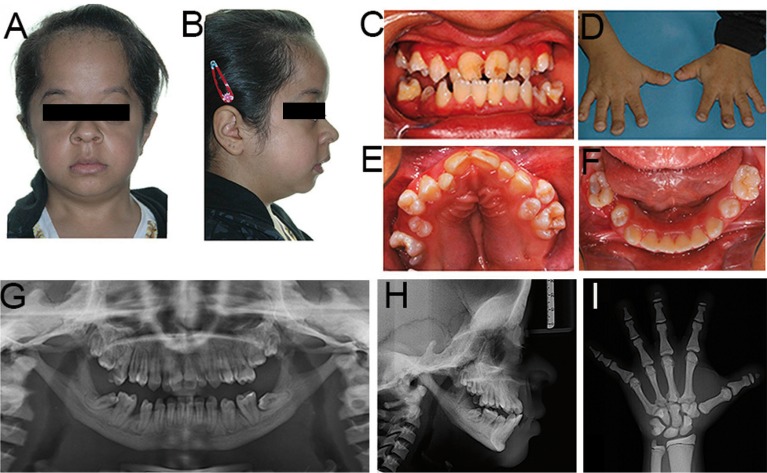
Extraoral (A and B) and intraoral photographs (C, E and F) and photograph of the hands (D). Panoramic radiograph (G), lateral cephalometric radiograph of the head (H), and radiograph of the left hand and wrist (I).

The patient exhibited clinical facial features of bilateral exophthalmos, facial asymmetry, frontal bossing, and micrognathia (Fig. 1A,B). Intraoral examination revealed mandible prognathism associated with an ogival palate, and generalized dental crowding, and typical enamel hypoplasia, with the presence of caries (Fig. 1C,E,F). Physical examination of the hands showed shortened fingers with spoon-shaped nails, giving a drumstich appearance (Fig. 1D). The patient did not show platybasia or any hearing problem, and the root canals were ample, and could be easily observed in the radiographs without atresic appearance. Based on these general and facial features, the diagnostic hypothesis was PYCD.

When asked about family history, the mother reported that there were no other relatives with similar clinical features. Panoramic, lateral cephalometric and hand-wrist radiographs, as well as computed tomography with 3D reconstruction, were requested for the assessment of bone characteristics. Micro-teeth, impacted teeth, missing teeth, and an obtuse mandibular angle almost horizontal in relation to the mandibular body were identified on the panoramic radiograph (Fig. 1G). Cephalometric radiography showed frontal bossing, an obtuse mandibular angle, and a horizontally positioned mandibular condyle, corresponding to Angle Class III malocclusion (Fig. 1H). The bone age determined from the hand-wrist radiograph (Fig. 1I) according to the Greulich-Pyle (11) method was 15 years. Evaluation of skeletal maturation according to the method of Martins and Sakima (12) showed that the patient was 2 to 3 years after peak growth, as demonstrated by union of the epiphysis of the proximal phalanx of the thumb and middle finger and partial union of the middle phalanx of the middle finger and terminal phalanxes and toes with drumstick appearance. Calcium and phosphorus levels were evaluated, and the laboratory values indicated no alterations. These same results were found regarding the markers for bone resorption (alkaline phosphatase and osteocalcin).

**Figure 2:**
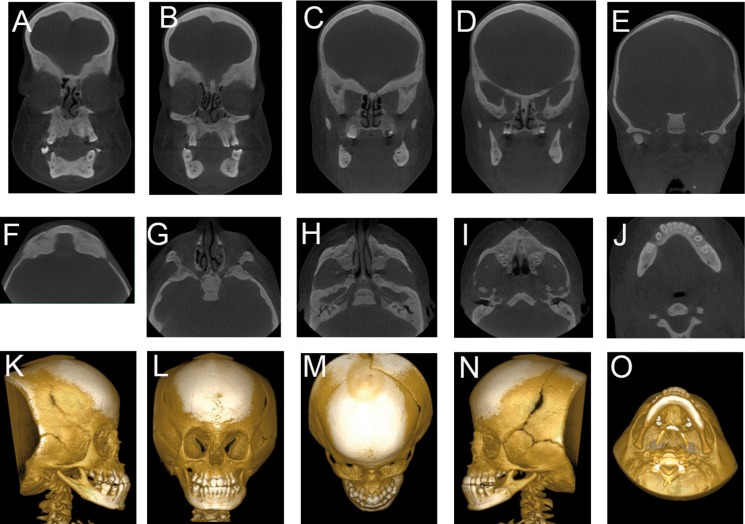
Coronal reconstructions of the skull in the anterior-posterior sequence (A, B, C, D and E), axial reconstructions in the upper-lower sequence (F, G, H, I and J), and 3D reconstructions (K, L, M, N, O and P).

Computed tomography showed frontal bossing, widening of the cranial and facial sutures, and open fontanelles (Fig. 2A-E), as well as hypoplasia of the maxillary sinuses and aplasia of the frontal and sphenoidal sinuses (Fig. 2F-J). The imaging scans showed dysplasia of the cranial, facial, hand and wrist bones (Fig. 2K-P). Taken together with the clinical findings, the radiographic features confirmed the diagnosis of PYCD.

## Discussion

Only few reports concerning PYCD describing the oral and maxillofacial features are available. So far, there is no specific protocol for PYCD bearers in dental clinics and its treatment is basically supportive, such as orthodontic therapy (6,7,13,14). According to Girbal *et al.* (15), osteomyelitis is a major risk, and can be faced as a serious complication of PYCD in patients who performed prior dental treatments. The justification is given by inappropriate remodeling of bone by dysfunctional osteoclasts, while bone formation continues normally, which leads to the formation of brittle bones and decreased vascularization which will continue to worsen with age. The increased susceptibility to osteomyelitis by the decreased number of osteons and marked obliteration of Haversians canals do jeopardize blood flow through the vessels, enhancing infection. Endosteum bone production will gradually increase with the aging process, narrowing the medullary spaces in the jaws and compromising vascularization and, as a consequence of such deficient blood flow; local immune competent cells diminish (15). Therefore, patients with PYCD are more likely to develop osteomyelitis than patients with normal bone (6,8,16).

There are reported cases of maxillary osteomyelitis following tooth extraction, leading to pathologic fractures in PYCD bearers. Normally, the patients show increased swelling and pus discharging in the involved region (17-19). Those who are affected with osteomyelitis, generally had no follow-up after dental management. In the case described in this paper, the patient attended to routine dental treatment. Her family was instructed to accomplish proper oral hygiene care, and to accomplish normal follow-up examination.

The life expectancy for PYCD bearers is considered normal, but bone fractures pose a primary threat to them with this disorder (13). Therefore, their life style should include special care as for what concerns avoiding risk factors in daily activities (14), since bone fractures caused by osteosclerosis is frequent in almost 50% of the cases, being, in most of the cases, transverse at the mids-haft of a long bone (7,8,20). The risk factors can be categorized depending on the activity the bearers have in their routine, with special remarks for the boys, who are more prone to radical sports. Nevertheless, mandibular fracture was seen only occasionally (8,21). During the anamnesis, the patient of this case reported a tibial fracture due to trauma. Such reports are typical of patients with PYCD, simply because minor traumas are indeed able to cause bone fractures, sometimes with serious outcomes (13,22).

The most evident clinical and radiographic feature of PYCD, however, is the presence of frontal, parietal and occipital bossing. Beaked nose, groove in midpalate, midfacial hypoplasia, mandibular hypoplasia and overcrowded teeth are almost always present. Other features are the narrow palate, presence of cross bite, and hypercementosis, with or without periodontal involvement due to the difficulty offered by the overcrowded teeth, which sometimes causes periodontitis. There have also been reports of supernumerary teeth, and narrowing of pulp chamber and root canals (7,13,23,24), which were not found in the present case. The patient in this study showed the traditional terminal phalanges of hands and toes with drumstick appearance, which were very remarkable (7). The clinical findings of the patient are in agreement with the current literature.

According to Xue *et al.* (25), the most commom phenotype of PYCD is short stature, present in 95.9% of the 97 previously reported cases, followed by increased bone density, present in 88.7% cases. Furthermore, PYCD exhibits similar clinical manifestations as adult-type osteopetrosis, including increased bone density and low bone turnover markers. We also observed in a patient these finds, corroborating with cases described by Alves *et al.* (7), and Pangrazio *et al.* (26).

In agreement to the Brazilian classification, a 13-year-old adolescent girl should have a height of 148 cm. The present patient was below this average, measuring 128 cm. Analysis of the hand and wrist radiograph according to the method of Greulich-Pyle (11) revealed a bone age of 15 years. This finding corresponded to skeletal maturation assessed by the method of Martins and Sakima (12), which indicated 2 to 3 years after peak growth. However, the biological age (13 years), bone age (15 years) and post-peak maturity would not favor orthodontic treatment. In addition, the region of the hand and wrist is related to other centers of skeletal growth and, thus, in this patient there would be no bone growth that favored an increase in stature or craniofacial growth. Computed tomography revealed open fontanelles compatible with the development of a child and not of an adolescent girl whose bone age was 15 years.

**Figure 3:**
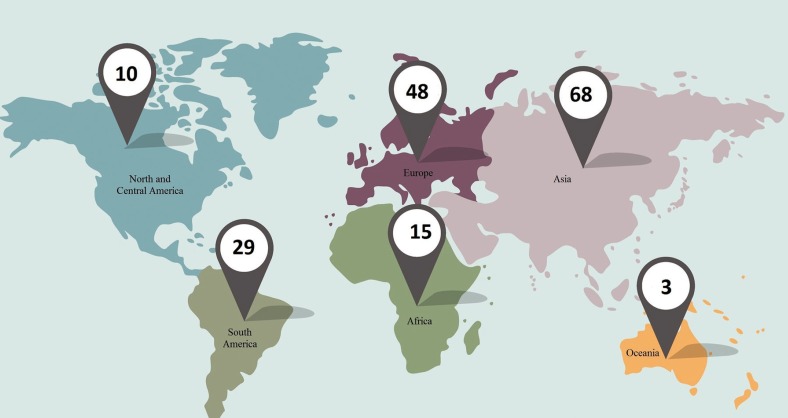
World distribution of the 173 cases of pycnodysostosis reported in the literature.

Articles on PYCD published from 1962 to March 2017 in English, Spanish and Portuguese were analyzed and the cases were mapped according to continent of occurrence of the disease. The Medline (PubMed) database was searched using the following keywords: pycnodysostosis; clinical features of pycnodysostosis; oral and maxillofacial pycnodysostosis features; pycnodysostosis dentofacial characteristics. Duplicate case reports, research articles, and articles without abstracts were excluded. A total of 271 articles were retrieved and 98 were excluded. The remaining 173 cases were selected according to the inclusion criteria and are indicated on the map (Fig. 3). As can be seen, most cases of this disorder were reported on the Asian (39.3%), European (27.7%) and South American (16.6%) continents. The most commonly affected country was Brazil, followed by India and Israel. Oral or maxillofacial findings of patients with PYCD were not reported in all cases.

The most mentioned characteristics of PYCD are cranial dysplasia, obtuse angle of the mandible, dysplasia of the clavicle, partial or total dysplasia of the terminal phalanges, and generally an increase in bone density (23). All of these were present in the clinical case reported. Additionally, cephalometric radiography revealed frontal bossing, an obtuse mandibular angle, and Angle Class III relationship. In the case of this type of maxillomandibular relationship, orthodontic treatment consisting of expansion of the maxilla and orthognathic surgery would be indicated for the correction of malocclusion (13,15). However, considering the dysplastic aspect of the bones, surgery would be limited since there is no consensus regarding the bone tissue response in patients with PYCD (2,6,9,10,13,15,27). The atypical horizontal insertion of the mandibular condyle in the glenoid fossa tends to project the mandible anteriorly, contributing to Angle Class III malocclusion.

Dental crowding and giroversions are generally indications for orthodontic treatment in patients with PYCD in an attempt to improve occlusion (13). However, the challenges are related to the difficulty in moving the teeth to an adequate position since bone resorption and bone formation are necessary events for appropriate orthodontic therapy. No protocol for orthodontic procedures in patients with PYCD is available and further studies are needed before the application of this type of treatment is possible. In this respect, the process of bone resorption and bone formation in patients with bone dysplasias such as PYCD should be better clarified, as well as orthopedic procedures for maxillary expansion (13,15,16,27).

Enamel hypoplasia, which is also generally present, plays an important role in oral health (2,7,8). Areas of brown enamel demineralization staining are prone to develop caries and appropriate care should be taken to prevent them. Good oral hygiene, clinical assessment, and periodic radiographic examinations are recommended (6-9).

Pycnodysostosis resembles osteopetrosis and the differential diagnosis is necessary. Consanguinity seems to play a role in these disorders. However, differences exist between both diseases. Osteopetrosis is an autosomal dominant disease, while PYCD is an autosomal recessive disease. Patients with osteopetrosis generally exhibit anemia, hepatosplenomegaly and immune deficiencies, while these characteristics are not observed in PYCD (17).

## Conclusions

Despite advanced genetic and biological knowledge, the diagnosis of PYCD continues to be based on clinical and radiographic features. The therapeutic approach should prevent complications and fracture injuries. With respect to oral and maxillofacial findings, good oral hygiene, clinical assessment and periodic radiographic examinations are recommended to improve occlusion and patient esthetics.

## References

[1] Terrazas K, Dixon J, Trainor PA, Dixon MJ (2017). Rare syndromes of the head and face: mandibulofacial and acrofacial dysostoses. Wiley Interdiscip Rev Dev Biol.

[2] Elmore SM (1967). Pycnodysostosis: A review. J Bone Joint Surg Am.

[3] Bonafe L, Cormier-Daire V, Hall C, Lachman R, Mortier G, Mundlos S (2015). Nosology and classification of genetic skeletal disorders: 2015 revision. Am J Med Genet A.

[4] Gelb BD, Shi GP, Chapman HA, Desnick RJ (1996). Pycnodysostosis, a lysosomal disease caused by cathepsin K deficiency. Science.

[5] Wen X, Yi LZ, Liu F, Wei JH, Xue Y (2016). The role of cathepsin K in oral and maxillofacial disorders. Oral Dis.

[6] Alves Pereira D, Berini Aytés L, Gay Escoda C (2008). Pycnodysostosis. A report of 3 clinical cases. Med Oral Patol Oral Cir Bucal.

[7] Alves N, Cantín M (2014). Clinical and radiographic maxillofacial features of pycnodysostosis. Int J Clin Exp Med.

[8] Muto T, Michiya H, Taira H, Murase H, Kanazawa M (1991). Pycnodysostosis. Report of a case and review of the Japanese literature, with emphasis on oral and maxillofacial findings. Oral Surg Oral Med Oral Pathol.

[9] Khoja A, Fida M, Shaikh A (2015). Pycnodysostosis with Special Emphasis on Dentofacial Characteristics. Case Rep Dent.

[10] Kamak H, Kamak G, Yavuz I (2012). Clinical, radiographic, diagnostic and cephalometric features of pycnodysostosis in comparison with Turkish cephalometric norms: A case report. Eur J Dent.

[11]  Greulich  WW,  Pyle  SI (1959). . Radiographic Atlas of Skeletal Development of the Hand and Wrist.

[12] Martins JCR, Sakima T (1977). Considerações sobre a previsão do surto de crescimento puberal. Ortodontia.

[13] Mujawar Q, Naganoor R, Patil H, Thobbi AN, Ukkali S, Malagi N (2009). Pycnodysostosis with unusual findings: a case report. Cases J.

[14] Ortegosa MV, Bertola DR, Aguena M, Passos-Bueno MR, Kim CA, de Faria ME (2014). Challenges in the orthodontic treatment of a patient with pycnodysostosis. Cleft Palate Craniofac J.

[15] Girbal I, Nunes T, Medeira A, Bandeira T (2013). Pycnodysostosis with novel gene mutation and severe obstructive sleep apnoea: management of a complex case. BMJ Case Rep.

[16] Rojas PI, Niklitschek NE, Sepúlveda MF (2016). [Multiple long bone fractures in a child with pycnodysostosis. A case report]. Arch Argent Pediatr.

[17] Kamat S, Sankar K, Eswari NJ, Gahlawat V, Jude BN, Negi A (2015). Management of chronic suppurative osteomyelitis in a patient with pycnodysostosis by intra-lesional antibiotic therapy. J Nat Sci Biol Med.

[18] Frota R, Linard RA, de Oliveira e Silva ED, Antunes AA, Carvalho RW, Santos Tde S (2010). Mandibular osteomyelitis and fracture in a patient with pyknodysostosis. J Craniofac Surg.

[19] Kato H, Matsuoka K, Kato N, Ohkubo T (2005). Mandibular osteomyelitis and fracture successfully treated with vascularised iliac bone graft in a patient with pycnodysostosis. Br J Plast Surg.

[20] Barnard B, Hiddema W (2012). Pycnodysostosis with the focus on clinical and radiographic findings. S Afr J Rad.

[21] Kirita T, Sugiura T, Horiuchi K, Morimoto Y, Yazima H, Sugimura M (2001). Mandibular reconstruction using a vascularised fibula osteocutaneous flap in a patient with pyknodysostosis. Br J Plast Surg.

[22] Song HK, Sohn YB, Choi YJ, Chung YS, Jang JH (2017). A case report of pycnodysostosis with atypical femur fracture diagnosed by next-generation sequencing of candidate genes. Medicine (Baltimore).

[23] Maroteaux P, Lamy M (1962). [Pyknodysostosis]. Presse Med.

[24] Fleming KW, Barest G, Sakai O (2007). Dental and facial bone abnormalities in pyknodysostosis: CT findings. AJNR Am J Neuroradiol.

[25] Xue Y, Cai T, Shi S, Wang W, Zhang Y, Mao T (2011). Clinical and animal research findings in pycnodysostosis and gene mutations of cathepsin K from 1996 to 2011. Orphanet J Rare Dis.

[26] Pangrazio A, Puddu A, Oppo M, Valentini M, Zammataro L, Vellodi A (2014). Exome sequencing identifies CTSK mutations in patients originally diagnosed as intermediate osteopetrosis. Bone.

[27] Hernández-Alfaro F, Arenaz Búa J, Serra Serrat M, Mareque Bueno J (2011). Orthognathic surgery in pycnodysostosis: a case report. Int J Oral Maxillofacial Surg.

